# 中国成人急性髓系白血病（非急性早幼粒细胞白血病）诊疗指南（2021年版）

**DOI:** 10.3760/cma.j.issn.0253-2727.2021.08.001

**Published:** 2021-08

**Authors:** 中华医学会血液学分会白血病淋巴瘤学组

**修订要点：**

• 增加推荐等级，证据等级按牛津大学EBM中心（Oxford Centre for Evidence-based Medicine Levels of Evidence）关于文献类型的五级标准。

• WHO分型中增加了胚系易感急性髓系白血病（AML），因此在病史采集及重要体征中，增加有无白血病或肿瘤家族史。

• 在遗传学分组中，根据等位基因比判断FLT3-ITD的预后意义；NPM1及FLT3-ITD共存时归入预后中等组；C-kit突变的预后意义限于D816位点突变。

• 增加MRD用于指导分层治疗。

• 异基因造血干细胞移植后，增加维持治疗内容。

• 调整老年AML标准剂量诱导治疗方案。

• 老年AML巩固治疗后，增加维持治疗内容。

• 低强度治疗中，去甲基化药物增加了阿扎胞苷，方案中增加了维奈克拉联合去甲基化药物。

**第一部分初诊患者入院检查、诊断**

一、病史采集及重要体征

• 年龄

• 此前有无血液病史［主要指骨髓增生异常综合征（MDS）、骨髓增殖性肿瘤（MPN）等］

• 是否为治疗相关性（包括肿瘤放疗、化疗）

• 有无重要脏器功能不全（主要指心、肝、肾功能等）

• 有无髓外浸润［主要指中枢神经系统白血病（CNSL）、皮肤浸润、髓系肉瘤］

• 有无白血病或者肿瘤家族史，有无遗传代谢性疾病病史

二、实验室检查

• 血常规、血生化、出凝血检查

• 骨髓细胞形态学（包括细胞形态学、细胞化学、组织病理学）

• 免疫分型

• 细胞遗传学（染色体核型），必要时荧光原位杂交（FISH）

• 分子学检测

PML-RARα、RUNX1-RUNX1T1、CBFβ-MYH11、MLL重排、BCR-ABL1、C-kit、FLT3-ITD、NPM1、CEBPA、TP53、RUNX1（AML1）、ASXL1、IDH1、IDH2、DNMT3a基因突变，这些检查是急性髓系白血病（AML）分型、危险度分层及指导治疗方案的基础[1]–[4]（证据等级1a）。

TET2及RNA剪接染色质修饰基因（SF3B1、U2AF1、SRSF2、ZRSR2、EZH2、BCOR、STAG2）突变等AML相关基因突变，这些检查对于AML的预后判断及治疗药物选择具有一定的指导意义[5]（证据等级2a）。

• 对于有CEBPA、RUNX1、DDX41等基因突变的患者，建议进行体细胞检查除外胚系易感AML

• 有意愿行异基因造血干细胞移植的患者行HLA配型

三、诊断、分类

AML的诊断标准参照WHO 2016造血和淋巴组织肿瘤分类标准，外周血或骨髓原始细胞≥20％是诊断AML的必要条件。但当患者被证实有克隆性重现性细胞遗传学异常t（8;21）（q22；q22）、inv（16）（p13q22）或t（16；16）（p13；q22）以及t（15；17）（q22；q12）时，即使原始细胞<20％，也应诊断为AML[3]。

四、AML的预后和分层因素

1. AML不良预后因素：

• 年龄≥60岁

• 此前有MDS或MPN病史

• 治疗相关性/继发性AML

• 高白细胞（WBC≥100×10^9^/L）

• 合并CNSL

• 合并髓外浸润（除外肝、脾、淋巴结受累）

2. 细胞遗传学/分子遗传学指标危险度分级：目前国内主要是根据初诊时AML细胞遗传学和分子遗传学的改变进行AML遗传学预后分组，具体分组见表1[4],[6]–[9]。

**表1:** 急性髓系白血病患者的预后危险度

预后等级	细胞遗传学	分子遗传学
预后良好	inv（16）（p13q22）或t（16；16）（p13；q22）	NPM1突变但不伴有FLT3-ITD突变，或者伴有低等位基因比
	t（8；21）（q22；q22）	FLT3-ITD突变^a^
		CEBPA双突变
预后中等	正常核型	inv（16）（p13；q22）或t（16；16）（p13；q22）伴有C-kit突变^b^
	t（9；11）（ p22；q23）	t（8;21）（q22；q22）伴有C-kit突变^b^
	其他异常	NPM1野生型但不伴有FLT3-ITD突变，或者伴有低等位基因
		比FLT3-ITD突变^a^（不伴有遗传学预后因素）
		NPM1突变伴有高等位基因比FLT3-ITD突变^a^
预后不良	单体核型	TP53突变
	复杂核型（≥3种），不伴有t（8；21）（q22；q22）、inv（16）（p13；q22）	RUNX1（AML1）突变^c^
	或t（16；16）（p13；q22）或t（15；17）（q22；q12）	ASXL1突变^c^
	−5	NPM1野生型伴高等位基因比FLT3-ITD突变^ac^
	−7	
	5q−	
	−17或abn（17p）	
	11q23染色体易位，除外t（9；11）	
	inv（3）（q21q26.2）或t（3；3）（q21q26.2）	
	t（6；9）（p23；q34）	
	t（9；22）（q34.1；q11.2）	

注：^a^低等位基因比为<0.5，高等位基因比为≥0.5。如没有进行FLT3等位基因比检测，FLT3-ITD阳性应按照高等位基因比对待。^b^C-kit D816突变对t（8;21）（q22；q22）、inv（16）（p13；q22）或t（16；16）（p13；q22）患者预后具有影响，其他的突变位点对预后没有影响，仍归入预后良好组。^c^这些异常如果发生于预后良好组时，不应作为不良预后标志。单体核型：两个或两个以上常染色体单体，或一个常染色体单体合并至少一个染色体结构异常。DNMT3a、RNA剪接染色质修饰基因突变（SF3B1、U2AF1、SRSF2、ZRSR2、EZH2、BCOR、STAG2）在不同时伴有t（8;21）（q22；q22）、inv（16）（p13q22）或t（16；16）（p13；q22）或t（15；17）（q22；q12）时，预后不良[5],[10]。但其循证医学证据级别不能等同于TP53、ASXL1、RUNX1等突变，暂不作为危险度分层的依据

**第二部分AML（非急性早幼粒细胞白血病）的治疗**

所有AML患者，在能够参加临床研究的情况下，均建议首选参加临床研究。在缺乏临床研究的条件下，可以参照下述建议进行治疗。可检测残留病（Measurable residual disease, MRD），既往称微小残留病（Minimal residual disease, MRD），可以采用多参数流式细胞术、PCR等方法进行检测。AML的治疗方案的选择主要根据患者对治疗的耐受性、遗传学危险度分层及治疗后的MRD进行动态调整。初诊不能耐受强烈治疗的患者经过低强度诱导治疗达完全缓解（CR）后，如果可以耐受强化疗，应按照可以耐受强化疗的患者进行治疗方案的选择。此外，在进行危险度分层时，除按照遗传学进行危险度分层外，近年还发现MRD在遗传学的基础上，对AML进行动态分层。对于MRD持续阳性，或者MRD由阴性转为阳性，尤其是巩固治疗完成后MRD阳性的患者，虽然遗传学分层属于预后中低危组，仍然建议进行造血干细胞移植。

一、年龄<60岁的AML患者

（一）诱导缓解治疗

1. 常规的诱导缓解治疗方案：标准剂量阿糖胞苷（Ara-C）100～200 mg·m^−2^·d^−1^×7 d联合去甲氧柔红霉素（IDA）12 mg·m^−2^·d^−1^×3 d或柔红霉素（DNR）60～90 mg·m^−2^·d^−1^×3 d[11]–[13]（证据等级1a）。

2. 含中剂量Ara-C的诱导治疗方案：高三尖杉酯碱（HHT）2 mg·m^−2^·d^−1^×7 d，DNR 40 mg·m^−2^·d^−1^×3 d，Ara-C前4 d为100 mg·m^−2^ ·d^−1^，第5、6、7天为1 g·m^−2^·12 h^−1^[9], [14]（证据等级1a）。

3. 其他诱导治疗方案：IA、DA、MA及HA＋蒽环类药物组成的方案，如HAA（HA+阿克拉霉素）、HAD（HA+DNR）等[7]。HHT（或三尖杉酯碱）联合标准剂量Ara-C的方案（HA）。化疗药物推荐使用剂量：标准剂量Ara-C 100～200 mg·m^−2^ ·d^−1^×7 d；IDA10～12 mg·m^−2^·d^−1^×3 d、DNR 45～90 mg·m^−2^·d^−1^×3 d、米托蒽醌（Mitox）6～10 mg·m^−2^·d^−1^×3 d、阿克拉霉素20 mg/d×7 d、HHT 2～2.5 mg·m^−2^·d^−1^×7 d（或4 mg·m^−2^·d^−1^×3 d）。临床工作中可以参照上述方案，具体药物剂量可根据患者情况调整。对于有严重合并症患者，参照老年不耐受强烈化疗患者的治疗方案。

（二）诱导治疗后监测

诱导治疗后恢复期（停化疗后第21～28天左右）复查骨髓以评价疗效，根据骨髓情况决定下一步的治疗方案。对于接受标准剂量，尤其是接受低强度诱导治疗的患者，可以在诱导治疗骨髓抑制期（停化疗后第7～14天左右）复查骨髓，可以根据骨髓原始细胞残留情况，调整治疗方案。

1. 标准剂量Ara-C诱导治疗后监测：

（1）停化疗后第7～14天复查骨髓：

①存在明显的残留白血病细胞（≥10％），可以考虑双诱导治疗，建议方案：

• 标准剂量Ara-C+蒽环或蒽醌类等药物（IDA或DNR、Mitox等）

• 含G-CSF的预激方案（如CAG方案：G-CSF+Ara-C+阿克拉霉素）

• 等待观察（尤其是处于骨髓增生低下的状态）

②残留白血病细胞<10％，但无增生低下：可给予双诱导治疗，采用标准剂量Ara-C+IDA或DNR、Mitox等；或等待恢复。

③增生低下，残留白血病细胞<10％：等待恢复。

（2）停化疗后第21～28天（骨髓恢复）复查骨髓、血象：①CR，进入缓解后治疗。②白血病细胞比例下降不足60％的患者，按诱导治疗失败对待。③未取得CR，但白血病细胞比例下降超过60％的患者可重复原方案1个疗程；也可换二线方案。④增生低下：残留白血病细胞<10％时，等待恢复；残留白血病细胞≥10％时，可考虑下一步治疗（参考双诱导治疗的方案或按诱导治疗失败患者选择治疗方案）。

2. 含中大剂量Ara-C方案的诱导治疗后监测：停化疗后第21～28天（骨髓恢复）复查骨髓、血象。①CR，进入缓解后治疗。②骨髓已恢复，但未达到CR标准的，按诱导治疗失败对待。③增生低下：残留白血病细胞<10％时，等待恢复；残留白血病细胞≥10％时，按治疗失败对待。

（三）CR后治疗的选择

按遗传学预后危险度分层治疗；蒽环类药物的剂量同诱导治疗方案。

1. 预后良好组：

（1）多疗程的大剂量Ara-C：大剂量Ara-C（3 g·m^−2^·12 h^−1^，6个剂量），3～4个疗程，单药应用[15]–[16]（证据等级1a）。

（2）其他缓解后治疗方案：①中大剂量Ara-C（1～2 g·m^−2^·12 h^−1^，6个剂量）为基础的方案：与蒽环/蒽醌类、氟达拉滨等联合应用，2～3个疗程后行标准剂量化疗，总的缓解后化疗周期≥4个疗程[7],[9],[14],[17]（证据等级1b）。② 2～3个疗程中大剂量Ara-C为基础的方案巩固治疗，继而行自体造血干细胞移植[18]–[20]（证据等级1b）。③标准剂量化疗（Ara-C联合蒽环/蒽醌类、HHT、鬼臼类等），总的缓解后化疗周期≥6个疗程或标准剂量化疗巩固3～4个疗程后行自体造血干细胞移植[21]（证据等级2b）。

2. 预后中等组：

（1）异基因造血干细胞移植。寻找供者期间行1～2个疗程的中大剂量Ara-C为基础的化疗或标准剂量化疗[22]（证据等级1a）。

（2）多疗程的大剂量Ara-C。大剂量Ara-C（3 g·m^−2^·12 h^−1^，6个剂量），3～4个疗程，单药应用[15]–[16]（证据等级1a）。

（3）2～3个疗程中大剂量Ara-C为基础的巩固治疗后行自体造血干细胞移植[18]–[20]（证据等级1b）。

（4）其他巩固治疗方案：①中大剂量Ara-C（1～2 g·m^−2^·12 h^−1^，6个剂量）为基础的方案：与蒽环/蒽醌类等药物联合应用，2～3个疗程后行标准剂量化疗，总的缓解后化疗周期≥4个疗程[7],[9],[14],[17]（证据等级1b）。②标准剂量化疗（Ara-C联合蒽环/蒽醌类、HHT、鬼臼类等），总的缓解后化疗周期≥6个疗程或标准剂量化疗巩固3～4个疗程后行自体造血干细胞移植[21]（证据等级2b）。

3. 预后不良组：

（1）尽早行异基因造血干细胞移植。寻找供者期间行1～2个疗程的中大剂量Ara-C为基础的化疗或标准剂量化疗[22]（证据等级1a）。

（2）无条件移植者予大剂量Ara-C（3 g·m^−2^·12 h^−1^，6个剂量），3～4个疗程，单药应用[15]–[16]（证据等级1a）。

（3）其他巩固治疗方案：① 2～3个疗程的中大剂量Ara-C为基础的化疗，或标准剂量化疗巩固，继而行自体造血干细胞移植[18]–[20]（证据等级1b）。②标准剂量化疗巩固（≥6个疗程）[21]（证据等级1a）。

4. 未进行染色体核型等检查、无法进行危险度分层者：参考预后中等细胞遗传学或分子异常组患者治疗。若诊断时WBC≥100×10^9^/L，则按预后不良组治疗（证据等级5）。

5. 异基因造血干细胞移植后，视复发风险及造血重建状态，FLT3-ITD阳性患者可以选择FLT3抑制剂进行维持治疗，其他患者可以选择去甲基化药物维持治疗[23]–[25]（证据等级1b）。

二、年龄≥60岁的AML患者

（一）年龄≥60～75岁患者的诱导治疗

1. 适合接受强化疗的患者（根据年龄、PS评分及合并基础疾病判断）：治疗前应尽量获得遗传学结果，根据患者的预后可以分为两种情况[26]。

（1）没有不良预后因素（a.不良遗传学异常；b.前期血液病病史；c.治疗相关AML）：对于治疗前没有获得遗传学结果的患者，治疗原则可以参照没有不良预后因素的情况。

①标准剂量化疗：标准剂量Ara-C（100 mg·m^−2^·d^−1^×7 d）联合IDA（10～12 mg·m^−2^·d^−1^）或DNR（45～60 mg·m^−2^·d^−1^）[27]–[30]（证据等级1a）。

②低强度化疗方案，具体方案见具有不良预后因素患者的低强度化疗方案（证据等级2c）。

（2）具有不良预后因素（a.不良遗传学异常；b.前期血液病病史；c.治疗相关AML）：

①低强度化疗：维奈克拉（100 mg，第1天；200 mg，第2天；400 mg，第3～28天）联合阿扎胞苷（75 mg·m^−2^·d^−1^，7 d）或地西他滨（20 mg·m^−2^·d^−1^，5 d）[31]–[33]（证据等级2a）。

阿扎胞苷（75 mg·m^−2^·d^−1^，7 d）或地西他滨（20 mg·m^−2^·d^−1^，5 d）[34]–[36]（证据等级2a）。小剂量化疗±G-CSF（如小剂量Ara-C为基础的方案：CAG、CHG、CMG等，C-阿糖胞苷、A-阿克拉霉素、H-高三尖杉酯碱、M-米托蒽醌）；阿扎胞苷或地西他滨联合小剂量化疗等[37]–[38]（证据等级2b）。

②标准剂量化疗：标准剂量Ara-C（100 mg·m^−2^·d^−1^×7 d）联合IDA（10～12 mg·m^−2^·d^−1^）或DNR（45～60 mg·m^−2^·d^−1^）[27]–[30]（证据等级2a）。

2. 不适合强化疗的患者：

（1）低强度化疗：维奈克拉（100 mg，第1天；200 mg，第2天；400 mg，第3～28天）联合阿扎胞苷（75 mg·m^−2^·d^−1^，7 d）或地西他滨（20 mg·m^−2^ ·d^−1^，5 d）[31]–[33]（证据等级1a）。

阿扎胞苷（75 mg·m^−2^ ·d^−1^，7 d）或地西他滨（20 mg·m^−2^·d^−1^，5 d）[34]–[36]（证据等级1a）。阿扎胞苷或地西他滨联合小剂量化疗；小剂量化疗±G-CSF（如小剂量AraC为基础的方案：CAG、CHG、CMG等）[37]–[38]（证据等级2b）。

（2）支持治疗。

（二）年龄≥75岁或<75岁且合并严重非血液学合并症患者的治疗

1. 低强度化疗：维奈克拉（100 mg，第1天；200 mg，第2天；400 mg，第3～28天）联合阿扎胞苷（75 mg·m^−2^·d^−1^，7 d）或地西他滨（20 mg·m^−2^ ·d^−1^，5 d）[31]–[33]（证据等级1a）。

阿扎胞苷（75 mg·m^−2^ ·d^−1^，7 d）或地西他滨（20 mg·m^−2^·d^−1^，5 d）[34]–[36]（证据等级1a）。阿扎胞苷或地西他滨联合小剂量化疗。小剂量化疗±G-CSF（如小剂量Ara-C为基础的方案：CAG、CHG、CMG等）[37]–[38]（证据等级2b）。

2. 支持治疗。

（三）强诱导化疗后骨髓情况监测及对策

停化疗后第21～28天复查骨髓、血象：① CR，进入缓解后治疗。②白血病细胞比例下降不足60％的患者，按诱导失败对待。③未达CR，但白血病细胞比例下降超过60％的患者，可重复原方案1个疗程；或更换二线方案。④增生低下：残留白血病细胞<10％时，等待恢复；残留白血病细胞≥10％时，按诱导治疗失败对待。

（四）CR后的治疗选择

1. 经过标准剂量诱导化疗达CR：

（1）标准剂量Ara-C（75～100 mg·m^−2^·d^−1^×5～7 d）为基础的方案巩固强化治疗。可与蒽环或蒽醌类（IDA、DNR或Mitox等）、HHT、鬼臼类等联合。总的缓解后化疗周期4～6个疗程[28]（证据等级2b）。

（2）年龄<70岁，一般状况良好、肾功能正常（肌酐清除率≥70 ml/min）、预后良好核型或伴有预后良好分子遗传学异常的正常核型患者可接受Ara-C 0.5～2 g·m^−2^·12 h^−1^×4～6个剂量，1～2个疗程。后改为标准剂量方案治疗，总的缓解后治疗周期4～6个疗程[15],[28]（证据等级2a）。

（3）年龄<70岁，一般状况良好、重要脏器功能基本正常、伴有预后不良因素、有合适供者的患者，可采用非清髓预处理的异基因造血干细胞移植治疗[39]–[40]（证据等级2a）。

（4）去甲基化药物（如阿扎胞苷或地西他滨）治疗，直至疾病进展[34]–[36]（证据等级2b）。

2. 经过低强度诱导化疗达CR：对于一些预后良好，达到CR后，能够耐受标准剂量化疗的患者，可以按经过标准剂量诱导化疗达CR的患者处理。也可以继续前期的低强度治疗方案。

（1）维奈克拉（400 mg，第1～28天）联合阿扎胞苷（75 mg·m^−2^·d^−1^，7 d）或地西他滨（20 mg·m^−2^·d^−1^，5 d），直至疾病进展[31]–[33]（证据等级1a）。

（2）阿扎胞苷（75 mg·m^−2^·d^−1^，7 d）或地西他滨（20 mg·m^−2^·d^−1^，5 d），直至疾病进展[34]–[36]（证据等级1a）。阿扎胞苷或地西他滨联合小剂量化疗；小剂量化疗±G-CSF（如小剂量Ara-C为基础的方案：CAG、CHG、CMG等）[37]–[38]（证据等级2b）。

3. 维持治疗：经过诱导和巩固治疗后，患者可用去甲基化药物（如阿扎胞苷或地西他滨）进行维持治疗，直至疾病进展[41]–[42]（证据等级1b）。

三、AML患者CNSL的预防和治疗

AML患者CNSL的发生率远低于急性淋巴细胞白血病（ALL），一般不到3％。参考NCCN的意见，在诊断时对无症状的患者不建议行腰椎穿刺（腰穿）检查。有头痛、精神混乱、感觉异常的患者应先行放射学检查（CT/MRI），排除神经系统出血或肿块。这些症状也可能是由于白细胞淤滞引起，可通过白细胞分离等降低白细胞计数的措施解决。若体征不清楚、无颅内出血的证据，可在纠正出凝血紊乱和血小板支持的情况下行腰穿。脑脊液中发现白血病细胞者，应在全身化疗的同时鞘注Ara-C（40～50 mg/次）和（或）甲氨蝶呤（MTX，5～15 mg/次）+地塞米松（5～10 mg/次）。若症状持续存在，脑脊液无异常，应复查。

（一）诊断时有神经系统症状的患者

首先应进行CT/MRI检查，除外出血或肿块。

1. 没有发现颅内/脊髓肿块者，进行腰穿：①脑脊液正常者：观察。如果症状持续存在，可以再次腰穿。②脑脊液发现白血病细胞者：鞘注化疗药物（2次/周）直至脑脊液正常，以后每周1次×4～6周。

2. 发现颅内/脊髓肿块或颅压增高者，建议先行放射治疗；然后鞘注药物（2次/周）直至脑脊液正常，以后每周1次×4～6周。

（二）无神经系统症状且第1次CR（CR_1_）后腰穿筛查脑脊液发现白血病细胞者

鞘注化疗药物2次/周，直至脑脊液恢复正常，以后每周1次×4～6周。若患者接受大剂量Ara-C治疗，应于治疗完成后复查脑脊液（证实脑脊液正常）；也可以配合腰穿、鞘注，至脑脊液恢复正常。

（三）无神经系统症状且CR_1_后腰穿筛查脑脊液正常者

已达CR的患者，建议行腰穿、鞘注，以进行CNSL的筛查。无CNSL患者建议进行4次鞘注治疗。尤其是治疗前WBC≥40×10^9^/L或单核细胞白血病（M_4_和M_5_）、t（8;21）/RUNX1-RUNX1T1、inv（16）白血病患者。

四、特别说明

在AML的整个治疗过程中应特别注意化疗药物的心脏毒性问题，注意监测心功能（包括心电图、心肌酶、超声心动图等）。DNR的最大累积剂量550 mg/m^2^。对于活动性或隐匿性心血管疾病、目前或既往接受过纵隔/心脏周围区域的放疗、既往采用其他蒽环类或蒽二酮类药物治疗、同时使用其他抑制心肌收缩功能的药物或具有心脏毒性的药物如曲妥珠单抗等情况，累积剂量一般不超过400 mg/m^2^。IDA的最大累积剂量290 mg/m^2^，Mitox的最大累积剂量160 mg/m^2^。计算累积剂量时还应考虑整个治疗周期的持续时间、同类药物（如不同的蒽环类药物）的使用情况。
